# Spatial–Temporal Variations in Parasitological Prevalence and Host-Related Risk Factors of Camel Trypanosomiasis and Its Vectors in North Eastern Kenya: A Repeated Cross-Sectional Study

**DOI:** 10.1155/2023/7218073

**Published:** 2023-04-28

**Authors:** Kennedy O. Ogolla, Judith K. Chemuliti, Florence N. Wamwiri, Joanna E. Auma, Richard K. Kurgat, Kennedy B. Wanjala, Lawrence G. Mugunieri, Phylis M. Alusi, Raymond E. Mdachi, Phoebe W. Mukiria, Sylvance O. Okoth

**Affiliations:** ^1^Biotechnology Research Institute, KALRO, P.O. Box 362-00902 Kikuyu, Kenya; ^2^East African Science and Technology Commission (EASTECO)/East African Community, Kigali, Rwanda

## Abstract

Camel trypanosomiasis (*Surra*) is endemic in the Horn of Africa. Understanding the spatiotemporal variations in *Surra* prevalence, vector dynamics, and host-related risk factors is important in developing effective control strategies. A repeated cross-sectional study was conducted to determine the *Surra* parasitological prevalence, livestock reservoirs, vector density/diversity, and host-related risk factors in Kenya. Random samples of 847, 1079, and 824 camels were screened at the start of the dry season, peak dry season, and during the rainy season, respectively. Blood samples were examined using the dark ground/phase contrast buffy-coat technique, and *Trypanosoma* species were identified based on their movement and morphology in wet and stained thin smears. Reservoir status for *Trypanosoma evansi* was assessed in 406 cattle and 372 goats. A rainy and dry seasons entomological surveys were conducted to determine the *Surra* vector abundance/diversity and spatiotemporal density changes. *Surra* prevalence was 7.1%, 3.4%, and 4.1% at the start of the dry season, peak dry season, and rainy season, respectively. Camel co-infections by *Trypanozoon (T. evansi* or *Trypanosoma brucei brucei*) and *Trypanosoma vivax* were recorded. Spatial variations in *Surra* prevalence were recorded at the beginning of dry (*X*_(7, *N* = 846)_^2^ = 110.9, *p* ≤ 0.001), peak dry (*X*_(7, *N* = 1079)_^2^ = 42.2, *p* ≤ 0.001), and rainy (*X*_(7, *N* = 824)_^2^ = 29.1, *p* ≤ 0.001) seasons. The screened cattle and goats tested negative for *Trypanozoon (T. evansi* or *T. b. brucei*), while two cattle tested positive for *Trypanosoma congolense*. Biting fly catches were composed of a single species from *Tabanus*, *Atylotus*, *Philoliche*, *Chrysops,* and *Stomoxys* genera. The total catches for *Philoliche*, *Chrysops*, and *Stomoxys* were higher in the rainy than dry season consistent with the prevalence results. *Surra* remains an important camel disease in the region with its prevalence varying in space and time. Camel co-infections by *Trypanozoon (T. evansi* or *T. b. brucei*) and *T. vivax* necessitate proper diagnosis of suspected cases and targeted therapy.

## 1. Introduction

Camel *Surra* is an important disease of camels (*Camelus dromedarius*) in the camel ecosystem in the Horn of Africa. The disease affects camel productivity with a negative impact on food and nutrition security as well as the livelihoods of pastoralist communities living in the region [1, 2]. Improving camel health through sustainable control of *Surra* is increasingly viewed as one of the strategies for enhancing the livelihoods of camel keepers and the resilience of pastoral production systems in Kenya [3, 4]. *Surra* is an insidious protozoal infection caused by *Trypanosoma evansi* with a wide geographic and host distribution [5]. While there is a sporadic occurrence of acute forms that are often fatal, the disease mostly presents as a chronic infection characterized by non-pathognomonic clinical signs [5].

The *T. evansi* parasite is transmitted mechanically by biting flies, whose apparent density and distribution vary by season [6]. Several species of biting flies have been confirmed to transmit *T. evansi* either experimentally or in nature [7–10]. One of the most efficient tools for sampling biting flies is the NZI trap that captures not only greater numbers of tabanids but also a wider diversity of the same [11]. The efficacy of the NZI trap can be enhanced by the use of natural (e.g., urine from cattle) or synthetic odor attractants. The population dynamics of biting flies can be influenced by several factors including vegetative cover, micro-climatic conditions, host availability, and the presence/absence of control interventions. Some of these factors are subject to human influences that, as expected, vary by geographic location.

Previous empirical studies on the epidemiology of camel *Surra* reveal spatial heterogeneities in the disease occurrence [6, 12, 1]. The occurrence of *Surra* is associated with risk factors such as the abundance and distribution of arthropod vectors, susceptibility of hosts, pathogenicity of the parasite, and presence of reservoir hosts among others [5, 6]. However, the spatial patterns of the disease and their determinants remain little known across the different areas that are inhabited by different camel-keeping communities. In Kenya, for example, recent work undertaken in some of the camel-keeping areas shows differences in prevalence across different geographical areas, but the full extent of the disparities and the associated risk factors have not been fully elucidated. There are different camel-keeping ethnic groups residing in the camel ecosystem in Kenya, which include the Somalis, Gabbra, Borana, Turkana, and Rendille. These communities vary in their experiences and knowledge of camel husbandry and disease management and control. The Somalis, for example, are generally regarded as the “original” camel keepers based on their comparatively longer experience in camel keeping and owning higher populations of camels than the Turkana and Borana [13]. These differences in experiences and knowledge could explain some of the variations in the disease occurrence in the ecosystem but these have not been sufficiently investigated and documented.

Studies on the epidemiology of *Surra* and indeed most vector-borne infections are best understood through longitudinal studies that allow for observation of changes in risk factors in the target populations over a considerable length of time [14]. However, the high mobility of camel herds and the difficult terrain in camel-keeping ecosystems makes such studies prohibitively resource-intensive. Consequently, epidemiological studies on camel *Surra* and its vectors are more often than not cross-sectional and therefore limited in terms of their ability to fully elucidate how changes in risk factors influence disease and vector occurrence in space and time. Repeated cross-sectional sampling of camel populations and *Surra* vectors over time (season) and space (site to site) is one of the approaches that can be applied to circumvent the challenges of longitudinal studies. This approach, if well-structured and administered, has the added advantage of providing a more detailed observation of disease risk factors over a reasonably longer time than one-off cross-sectional studies.

A repeated cross-sectional sampling design over short time frames was used in the present study to assess the extent and nature of change in the disease prevalence, vectors, reservoirs, and host-related risk factors of camel *Surra* in eight selected sites inhabited by different ethnic communities in Marsabit and Isiolo counties.

## 2. Materials and Methods

### 2.1. Ethical Approval and Informed Consent

The study was approved by the Kenya Agricultural and Livestock Research Organization (KALRO)-Institutional Animal Care and Use Committee (KALRO-IACUC). The camel keepers, who participated in this study, gave informed consent before their camels were sampled.

### 2.2. Study Design, Study Locations, and Sites

**Figure 1:**
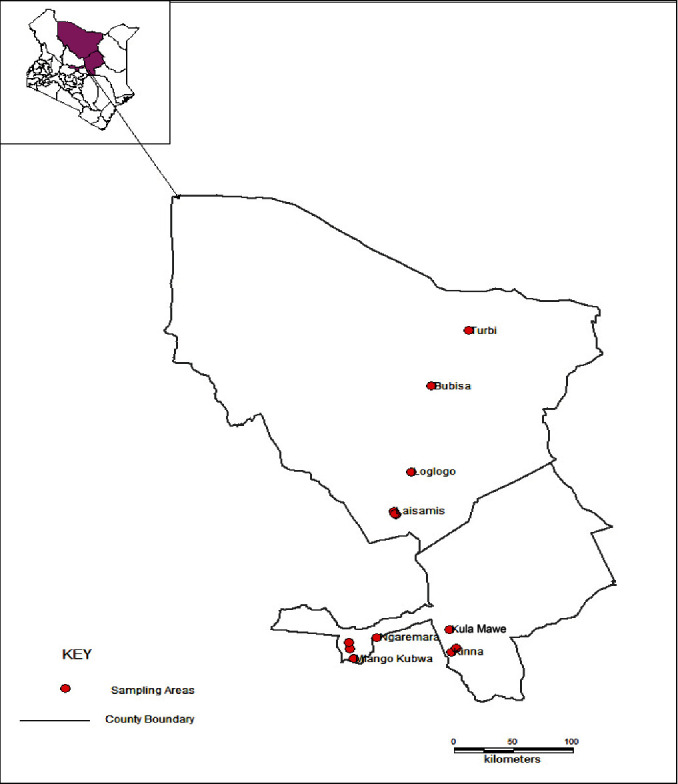
Map of Isiolo and Marsabit counties showing the study sites.

The study was carried out between September 2018 and April 2020 in eight pre-selected sites in Isiolo and Marsabit counties (four sites per county). Site selection was based on a cluster sampling technique guided by the geographic locations and ethnic formations. The study sites were Livestock Marketing Division (LMD), Ngaremara, Kinna, and Kula Mawe in Isiolo, and Turbi, Bubisa, Loglogo, and Laisamis in Marsabit (Figure 1).

Animal screening exercises were conducted at the beginning of the dry season (September–October 2018), at the peak of the dry season (March–April 2019), and during the rainy season (February–March 2020). All the animals sampled were from herds reared under an extensive pastoralist system. Any animal from selected herds that had not received trypanocide treatment in the last 3 months before the study met the inclusion criteria.

### 2.3. Selection of Camel, Sample Collection, and Analysis

A random sample of 847, 1079, and 824 camels of mixed ages and sex was screened for trypanosome parasites at the beginning of the dry season, peak dry season, and the rainy season, respectively. Blood was sampled from the jugular vein and examined using the dark ground/phase contrast buffy-coat technique (BCT) as described by Murray et al. [15]. Packed cell volume (PCV) was determined using Hawksley micro-hematocrit reader (Hawksley, Lancing, United Kingdom). Briefly, the animals were physically restrained, and blood samples were aseptically collected from the jugular vein into 5 ml vacutainer tubes laced with di-sodium salt of ethylene diamine tetra-acetate (EDTA). The blood was then transferred into heparinized capillary tubes, sealed on one end, and centrifuged at 12,000 rpm for 5 minutes [5], and the percentage hematocrit expressed as PCV recorded. The capillary tube was cut using a diamond pen 1 mm below the buffy coat to include the upper layer of red blood cells, and the buffy coat expelled up to 3 mm above to include plasma onto a clean microscope slide, covered with a cover slip, and examined for motile trypanosomes under a microscope magnification ×400 [5]. Trypanosome species were identified based on their movement and morphology as described by Desquesnes et al. [5]. Also, Giemsa-stained dry blood smears were prepared from this lot for identification of trypanosome species based on features described by Desquesnes et al. [5] for *T. evansi* and Osório et al. [16] for *Trypanosoma vivax*. The thin smears were fixed with absolute methanol for 1 minute and stained with Giemsa (10%) for 45 minutes, dried, and examined under oil immersion at ×1000 magnification [16]. Data on each animal's age, sex, and body condition score (BCS) were recorded. To assess the reservoir status of *T. evansi* in other livestock species, blood samples were aseptically collected from the jugular vein of 406 randomly selected cattle (drawn from 26 herds) and 372 goats (from 20 flocks) and screened during the rainy season. The body condition of all sampled animals was scored on a scale of 1–5 (where 1 was poor and 5 was excellent) for the three species through visual observation taking into account the extent to which ribs and spinous processes were exposed and degree of fat and muscle deposition in various places on the animal's body. Age categorization for each livestock species was as follows, viz., camels (adults = 5 years, young adults = 2–5 years, calves = 2 years) and cattle (adults = >2 years, young adults = >1–2 years, calves = <1 year). All sampling sites were geo-referenced using a hand-held Global Positioning System Garmin-GPSMAP® 76 (Garmin Ltd., Kansas, USA), and the points were used to generate a map.

### 2.4. Fly Trapping and Identification

Two surveys were carried out in September–October 2018 (at the beginning of the dry season) and in February–March 2020 (rainy season) using the NZI trap [17] in the same study sites, where animals were sampled. Traps were baited with acetone and cow urine dispensed from plastic bottles. The cow urine used had been aged at ambient temperatures for 2–4 weeks before the surveys to fully release the olfactory components. About 200 ml of urine was placed inside a 500 ml plastic container cut in half. Thus the urine was exposed to evaporation through an aperture of about 2 inches. On the other hand, about 25 ml acetone was placed into a 200 ml plastic bottle with a cap that had a 3 mm aperture. The odor dispensers were placed beside each other at ground level, approximately 30 cm in front of the trap entrance. Traps were set out in habitats that are considered suitable for fly infestation, viz., grazing and watering grounds. In each area, 10–15 traps were set out at intervals of about 200–400 m. The traps were left in position for 48 hours. All trapping sites were georeferenced. At the end of the trapping period, biting flies that were captured were identified, counted, and recorded. Individual flies were identified to the genus level using morphological parameters such as the shape of the head and wing coloration as previously described [18]. The flies were thereafter preserved in 70% ethanol for further processing in the laboratory. Pictures of the captured flies were taken using a DinoCapture® 2.0 digital microscope. During the trap-setting activities, unstructured observations of the surrounding environment and fly behavior were also made. Fly density was calculated as flies/trap/day (FTD).

### 2.5. Data Analysis

Data were coded, cleaned, and entered in Microsoft Excel 2016. Statistical analysis was carried out using the GenStat statistical analysis program (GenStat 15^th^ edition). Prevalence was calculated as the number of trypanosome-positive animal(s) divided by the total number of examined animals and then multiplied by 100 (with an assumption that the animals sampled had not received any prophylactic treatment and were thus all at risk). Descriptive statistics were computed, and results were presented as mean with their standard deviations (mean ± SD). An independent sample *t*-test was used to determine differences in the body condition scores and PCV of infected and non-infected and of male and female camels. Pearson Chi-square was used to detect statistical differences in trypanosome infection based on sex, age categories, sampling sites, body condition, and anemic status. Analysis of variance (ANOVA) was performed by one and two-way ANOVA to show the statistical differences, if any, in PCVs and BCS between the age categories and sampling sites. All significant levels were stated at *p* < 0.05. The PCV was considered anemic if it was ≤23 for camels (≤25 for cattle and goats) and normal if >23 for camels (>25 for cattle and goats), while BCS (BCS ≤ 2.5) was considered poor while (BCS > 2.5) was considered above average to good. The apparent fly density was expressed as the average number of flies caught per trap per day (FTD). The apparent density was calculated by dividing the total number of flies captured (*ΣF*) by the product of the number of functioning traps used to catch them (*T*) and the number of days for which the traps were operational (*D*) (FTD = *ΣF*/*T* × *D*). Traps that were not operational for some reason (knocked down by the wind, vandalized) that trap day were excluded from the sum of trap days.

## 3. Results

### 3.1. Characteristics of Animals Sampled

A total of 847, 1079, and 824 camels from different herds were screened at the beginning of the dry season, peak of the dry season, and the rainy season, respectively. The animals were drawn from 49, 59, and 52 herds at the beginning of the dry season, peak of the dry season, and the rainy season, respectively. In all the surveys, a bigger proportion of screened camels were females (71.0%, 79.9%, and 80% at the beginning of dry season, peak dry season, and rainy season screenings, respectively). Disaggregation of screened camels by sex and age categories in each study site is provided as Table A in the supplementary file.

### 3.2. Prevalence of Trypanosome Infection

The overall prevalence of trypanosomiasis based on the micro-hematocrit BCT and Giemsa-stained thin smear parasitological tests was 7.1% [CI: 5.5–9.0], 3.4% [CI: 2.4–4.7], and 4.1% [CI: 2.9–5.7] at the beginning of the dry season, peak dry season, and during the rainy season, respectively. The prevalence in Marsabit (10.4%) at the beginning of the dry season was almost thrice that in Isiolo County (*X*_(1, *N* = 846)_^2^ = 15.6, *p* = *p* ≤ 0.001). Similarly, Marsabit county (6.1%) recorded a significantly higher prevalence (*X*_(1, *N* = 1079)_^2^ = 24.8, *p* = *p* ≤ 0.001) compared to Isiolo (0.6%) at the peak of the dry season. There was a marginal difference in the prevalence of *Surra* recorded in Marsabit (4.8%, CI: 2.9–7.3) and Isiolo (3.5%, CI: 2.0–5.8) (*X*_(1, *N* = 824)_^2^ = 0.77, *p* = 0.38) during the rainy season. A higher prevalence was recorded in males in the three surveys (11.0%, 5.5%, and 5.5%) compared to females (5.5%, 2.9%, and 3.8%) at the beginning of the dry season, peak dry season, and in the rainy season, respectively. These differences in prevalence by sex were statistically significant at the beginning of the dry season (*X*_(1, *N* = 841)_^2^ = 7.9, *p* = 0.005), but not at the peak dry season (*X*_(1, *N* = 1079)_^2^ = 3.6, *p* = 0.06) and during the rainy season (*X*_(1, *N* = 824)_^2^ = 0.92, *p* = 0.34). Calves recorded the highest trypanosome prevalence (5.1%) in the rainy season followed by young adults (4.1%) and adults (3.7%). On the other hand, adults (3.6%) and young adults (5.0%) recorded the highest prevalence at the beginning of dry and peak dry seasons, respectively. The differences in prevalence by age were not significant in all three surveys. There were, however, significant variations in trypanosome prevalence recorded between the eight [8] sampling sites at the beginning of dry (*X*_(7, *N* = 846)_^2^ = 110.9, *p* = *p* ≤ 0.001), peak dry (*X*_(7, *N* = 1079)_^2^ = 42.2, *p* = *p* ≤ 0.001), and rainy (*X*_(7, *N* = 824)_^2^ = 29.1, *p* = *p* ≤ 0.001) seasons. A site like Kula Mawe recorded an increase in prevalence in the wet season screening (8.7%) compared to that recorded at the peak of the dry season (1.0%), while sites like Loglogo, Turbi, and Laisamis recorded a decrease in prevalence. No single case of trypanosome infection was recorded in LMD (0.0%) in all the surveys as shown in Table 1.

There were significant herd prevalence variations at the beginning of dry (*X*_(48, *N* = 846)_^2^ = 179.4, *p* = *p* ≤ 0.001), peak dry (*X*_(58, *N* = 1079)_^2^ = 133.2, *p* = *p* ≤ 0.001), and rainy (*X*_(51, *N* = 824)_^2^ = 242.3, *p* = *p* ≤ 0.001) seasons. The highest herd prevalence was recorded in Loglogo (71.4%) at the beginning of the dry season, followed by Kula Mawe (60.0%) in the rainy season, and finally Turbi (37.5%) at the peak of the dry season. Herd prevalence ranged from as low as 0.0% to as high as 71.4% as presented in Table 2.

### 3.3. Trypanosome Species Detected from Camel Blood Samples

**Figure 2:**
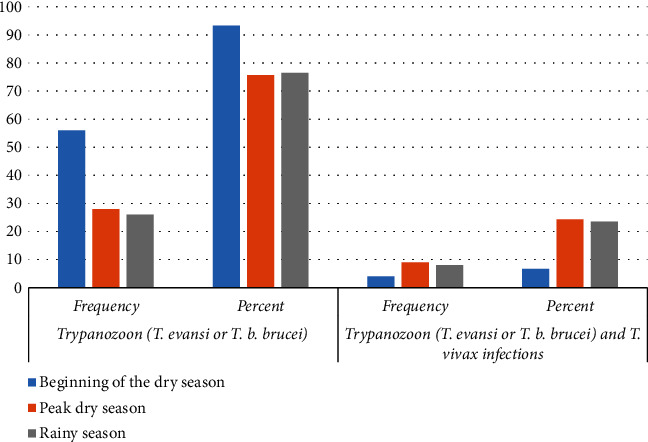
Trypanosome species that were isolated in the three sampling seasons in camels.

Single-species infections with *Trypanozoon* (*T. evansi* or *T. b. brucei*) were predominant although there were few cases of mixed infections of *Trypanozoon* (*T. evansi* or *T. b. brucei*) and *T. vivax* in the three surveys as shown in Figure 2.

### 3.4. Host-Related Risk Factors

Proportionately, fewer camels 6.8% (CI: 5.2–8.7) had severe anemia (PCV < 20) in the rainy season compared to 8.8% (CI: 7.2–10.7) and 8.2% (CI: 6.5–10.3) recorded at the peak of the dry season and the beginning of the dry season, respectively. In the three surveys, infected camels recorded a significantly lower mean PCV compared to non-infected camels at the beginning of dry [*t*(843) = 10.8, *p* = *p* ≤ 0.001], peak dry [*t*(1076) = 8.50, *p* = 0.001], and rainy [*t*(821) = 9.78, *p* = 0.001] seasons. Similarly, the three surveys revealed almost a direct correlation between severe anemia (low PCVs) and trypanosome infection. The mean PCVs of infected camels were 20.1 ± 4.3, 20.1 ± 3.17, and 19.4 ± 3.3 compared to 25.9 ± 4.0, 25.5 ± 3.8, and 25.2 ± 3.4 for non-infected camels at the beginning of the dry season, peak dry season, and during the rainy season, respectively. The mean PCVs of infected and non-infected camels in the three age categories are summarized in Table 3.

The overall anemia (PCV ≤ 23) prevalence in camels was 28.6% (CI: 25.5–31.8) in the rainy season. The proportion of anemic animals in Marsabit (28.3%; CI: 23.9–32.9) was almost equivalent to that recorded in Isiolo (28.8%; CI: 24.6–33.4). Of the infected camels during the rainy season, 91.2% (CI: 76.3–98.1) were anemic (PCV ≤ 23), while 83.3% (CI: 71.5–91.7) and 83.80% (CI: 68.0–93.8) of infected camels at the start of the dry and the peak of dry seasons were anemic indicating a higher proportion of infections in anemic animals. Moreover, there was a marked variation in anemia prevalence in the eight sampling sites in the wet season survey ((*X*_(1, *N* = 824)_^2^ = 33.8, *p* = 0.001) with Loglogo and Ngaremara recording the highest proportions of anemic camels at 46.0% (CI: 36.0–56.3) and 35.4% (CI: 27.7–43.7), respectively, consistent with trypanosome prevalence levels, while Bubisa (in Marsabit County) recorded the lowest prevalence (13.0%; CI: 7.1–21.2) of anemic animals. A significant proportion of anemic camels during the rainy season (86.8%; CI: 81.8–90.9), at the beginning of the dry season (77.5%; CI: 71.4–82.8), and at the peak of the dry season (89.6%; CI: 85.6–92.8) tested negative for trypanosomiasis on BCT and thin-stained smears. Of the 34 trypanosome-positive camels during the rainy season, three (8.8%) had PCV within the normal reference range for camels (PCV > 23%). Similarly, 10 (16.7%) camels of the 60 infected at the beginning of the dry season and 6 (16.2%) camels of the 37 infected at peak of dry season surveys had PCVs within the normal reference ranges (Table 4).

In the three surveys, non-infected camels recorded comparatively higher mean BCS of 3.3 ± 0.6, 3.1 ± 0.4, and 3.1 ± 0.5 compared to infected camels 2.8 ± 0.7, 3.0 ± 0.4, and 3.1 ± 0.5 at the beginning of the dry season, peak dry season, and during the rainy season, respectively.

### 3.5. Reservoir Status in Cattle and Goats

The majority of screened cattle were females (78.5%) compared to males (21.5%) of the *Bos indicus* breed. A sizeable proportion of screened cattle were adults (69.9%), followed by calves and young adults at 17.8% and 12.3%, respectively. Only two cows tested positive for *Trypanosoma congolense* by micro-hematocrit BCT and Giemsa-stained thin smear giving an overall trypanosome prevalence of 0.5% (CI: 0.1–1.8). Overall mean PCV in cattle was 28.9 ± 3.9 with Marsabit County recording an almost comparable mean PCV (29.6 ± 3.5) with Isiolo (28.3 ± 4.1) county. Of the screened cattle, 18.5% were anemic based on set anemia cut-off (PCV ≤ 25) in the present study. Isiolo County recorded a significantly higher proportion of anemic cattle (61.3%, (*X*_(1, *N* = 405)_^2^ = 4.23, *p* = 0.001) compared to Marsabit County (38.7%). Whereas there were slight variations in the proportion of anemic cattle recorded in the eight sampling sites, the differences were not statistically significant (Table B in the supplementary file). The two cows that tested positive for trypanosome parasites had normal PCV. Cattle in Marsabit county recorded a significantly higher [(*t*(349) = 27.6, *p* = 0.001)] mean BCS (3.8 ± 0.3) compared to those in Isiolo (2.9 ± 0.3), with the two trypanosome positive cattle in Isiolo recording above average BCS. All the goats screened tested negative for trypanosome parasites using the hematocrit centrifugation technique (buffy coat) and thin-stained smears.

### 3.6. Vector Density and Diversity

Cumulatively, 716 biting flies were captured in Marsabit County over 144 trap days, while 501 flies were captured in Isiolo county over 152 trap days. Whereas both *Tabanus* spp. and *Stomoxys calcitrans* were captured in all the locations sampled, catch composition was biased toward *Tabanus* spp. (more than 60%) in all trapping locations except in LMD where the numbers were almost equal. Inter-county comparison indicated a significant difference between the relative proportions of *Tabanus* and *Stomoxys* captured (*X*^2^ = 4.5, *p* = 0.03). The most common tabanids captured in the study belonged to the *Tabanus* and *Atylotus* genera, of which the most abundant species overall was *Atylotus agrestis*. A single species from each of five genera, viz., *Tabanus*, *Atylotus*, *Philoliche*, *Chrysops*, and *Stomoxys*, were captured. The catch distribution in Marsabit county was *Tabanus* 79.5%, *Philoliche* 1.1%, *Chrysops* 0.1%, and *Stomoxys* 19.3%. For Isiolo county, the total catch was distributed as follows: *Tabanus* 59.3%, *Philoliche* 14.4%, *Chrysops* 2.0%, and *Stomoxys* 24.4%. *Philoliche* spp. made up about 6% of the total catch. Interestingly, about 50% of all *Philoliche* were captured in Kula Mawe, Isiolo county. The very high number of *Tabanus* (*n* = 263) captured in only three dry season traps in Bubisa (Marsabit) is considered a site-specific anomaly, which may not be representative of the area. During both surveys, numerous camel flies *Hippobosca camelina* and *Haematobia* spp. were observed on and around the camels. However, these flies generally avoided the traps, and only one specimen was found in the traps. Additionally, two tsetse species *Glossina pallidipes* and *Glossina longipennis* were captured in Kinna, Isiolo county. Details of the fly species caught per site in the dry and rainy seasons are detailed in Tables 5 and 6.

### 3.7. Seasonal Variation of Apparent Fly Density

**Figure 3:**
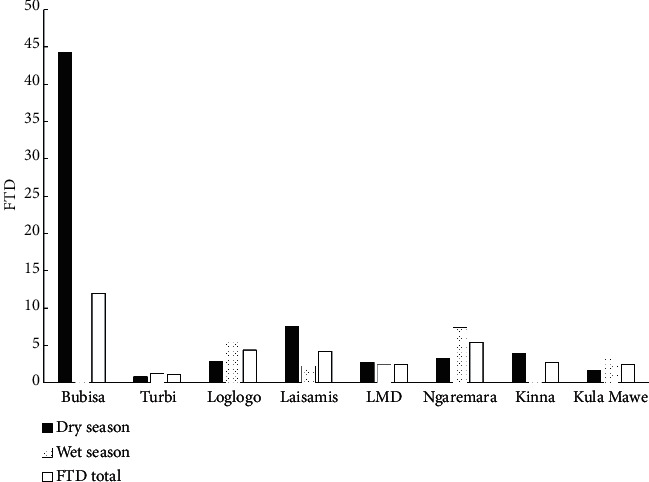
Average FTD values obtained per site in the wet and dry season surveys.

The present study did not identify species that were found exclusively in either the rainy or the dry season, perhaps due to the relatively low densities and diversity of the species captured. Considering the different fly species, the total catches for *Philoliche*, *Chrysops*, and *Stomoxys* were higher in the rainy season than in the dry season in all locations. In most locations, more flies were captured during the rainy season than in the dry season. Considering the total number of biting flies caught (both *Tabanus* and *Stomoxys*) and discounting the catches in Bubisa, the average number of flies caught per trap per day was slightly higher during the rainy season in both counties. Despite the overall trend in seasonal variation, site-level inconsistencies were observed in Laisamis and Kinna where the dry season FTDs were higher than the rainy season FTDs (Figure 3).

These results can be explained, in part, by locust and tsetse control interventions that were being carried out in Laisamis and Kinna, respectively, at the time of the survey. These operations, using insecticides with a non-specific effect, may have resulted in the decline in biting fly density during the rainy season.

## 4. Discussion

Temporal and spatial variation in *Surra* prevalence were demonstrated in the present study. The highest trypanosome prevalence was recorded at the beginning of the dry season followed by the wet season survey, while the lowest prevalence was recorded at the peak of the dry season. Seasonal fluctuations in *Surra* prevalence have been previously reported [1, 19]. The parasitological prevalence of *Surra* recorded in Isiolo county in the present study is comparable to those reported by Ngaira et al. [20] and Njiru et al. [21] in earlier studies conducted in Kenya and elsewhere [22–24]. While the present study detected both single-species trypanosome infection by members of the *Trypanozoon* (*T. evansi* or *T. b. brucei*) and mixed infections of *Trypanozoon* (*T. evansi* or *T. b. brucei*) and *T. vivax* in camels, previous studies have largely reported *T. evansi* infections in camels [25, 26], although sporadic *T. vivax* infections have been reported in recent studies [27, 28], indicating *T. vivax* is a potential risk to camel health. This calls for a change in the therapeutic management of *Surra* in camels, which is presently mostly targeted at and presumed to be caused by *T. evansi*. The relatively higher infection rates recorded in male camels compared to females in the present study contrast findings by previous studies [22, 29, 30]. The low prevalence in female camels may be attributed to better management accorded to females due to the economic value attached to them in terms of milk production in the region and may not be an indicator of reduced physiological susceptibility to trypanosomiasis. Male and female camels are known to have an almost equal risk of exposure to trypanosomiasis infection in areas with high biting flies challenge, especially when they are grazed together. However, camel keepers in the study area are known to graze lactating camels close to homesteads where the population of biting flies may be low and hence reduced risk of exposure. Earlier studies reported no sex-related increase in susceptibility to trypanosomiasis infection [22, 30]. However, an isolated study by Njiru et al. [31] reported a significantly higher prevalence in male camels compared to females in Kenya.

There was no significant difference in trypanosomiasis prevalence based on age in the present study. Camels of all ages seem to be at risk of being exposed to the biting flies when grazed or browsed together in infested regions and hence of coming down with the disease. Conversely, some studies have recorded a relatively higher prevalence in adults and young adults compared to calves [29, 31], which may be attributed to different management approaches accorded to the age categories. Njiru et al. [32] reported a relatively lower prevalence in suckling calves compared to adults, which was attributed to low exposure levels to biting flies and passive immunity from maternal antibodies. On the other hand, Lemecha et al. [33] found a higher prevalence in calves compared to the other age categories.

The present findings further established a clear spatial variation in the prevalence of *T. evansi* in the different geographic sites with some sampling sites recording high prevalence indicating defined *Surra* foci while others recorded very low prevalence. Whereas the highest trypanosomiasis prevalence was recorded in Loglogo, no single case of trypanosome infection in camels was detected in the LMD sampling site in the three seasons. This may be an indication that either the LMD area and/or the grazing areas of LMD camel keepers may not have biting flies or circulating trypanosome isolates or may point to better management practices and grazing patterns by the Somali ethnic community residing in the region. Indeed, our earlier survey on knowledge and trypanocidal use by the ethnic communities in the study area revealed that the Somali ethnic group residing in the LMD area were the most knowledgeable on camel *Surra* and recorded the highest prophylactic use of quinapyramine sulphate and chloride [34]. Nonetheless, the vector survey captured a few biting flies known to transmit trypanosome infection in the LMD region, although an assessment of fly infectivity was not carried out.

Herd prevalence variations were observed in the present study with the highest herd prevalence recorded in Loglogo (inhabited by the majority Rendille ethnic community) at the beginning of the dry season, followed by Kula Mawe (inhabited by the majority Borana ethnic community) during the rainy season, and finally Turbi (inhabited by the majority Gabbra ethnic community) at the peak of the dry season, which may be attributed to different *Surra* management practices by the communities as previously established [34]. Previous studies conducted in different localities in Somaliland and Ethiopia, both located in the Horn of Africa camel-keeping belt, reported comparable geographic [2, 26] and herd [22] variations in trypanosomiasis prevalence. Altitude-dependent variation in trypanosomiasis prevalence has also been reported [22]. The high prevalence of trypanosomiasis infection recorded in some herds in the present study may be attributed to either a high population of biting flies or concentrated infections in places where the herds are grazed. Camel husbandry practices vary across the ethnic communities inhabiting different locations in the present study with some known to manage their camels better than others [13, 34], which may also account to variations in *Surra* prevalence.

Anemia is almost a pathognomonic finding in trypanosomiasis cases [35, 36]. In all three surveys, infected camels had significantly lower mean PCV compared to non-infected camels, an indication of chronicity of *T. evansi* infection in camels [31]. The three surveys revealed an almost direct correlation between severe anemia and trypanosome infection, consistent with findings of numerous other studies in camels [27, 30].

In the present study, camels that had trypanosomiasis recorded a comparatively lower mean BCS compared to non-infected camels, further pointing to the chronicity of trypanosomiasis in camels. Loss of body condition (emaciation) is a typical sign reported in chronic cases of camel *Surra* [21]. Our finding, however, contrasts previous studies by Olani et al. [22] and Gerem et al. [26] that did not find any significant difference in mean BCS of infected and non-infected camels. Camels are likely to have poor BCS at peak dry season due to deterioration in forage quantity, quality, and the long distances the animals travel in search of pasture, all of which are stress-inducing, and the BCS are likely to improve during the wet season when conditions are better. The current findings revealed a lower mean BCS at peak of the dry season compared with that recorded at the beginning of the dry season. The poor BCS observed during the wet season survey may be because animals take time to recover from the weight lost during the dry season. The low BCS corresponded with the decrease observed in mean PCV of screened animals and even a lower mean PCV of trypanosome-positive animals.

The present study did not detect *T. evansi* in cattle and goats in all the sampling sites, even in cases where the animals were grazed together and shared watering points, which is consistent with the results of other studies [12, 37]. However, some studies have reported experimental [38] and natural [37] infections of goats with *T. evansi*. Similarly, natural *T. evansi* infections in cattle have been reported [27, 39, 40].

Both tabanids and *Stomoxys* were captured in the present entomological survey with tabanids being more abundant than *Stomoxys*. The preponderance of tabanids has been observed in a previous study [41]. In contrast, *Stomoxys* was the most abundant fly species in a study conducted in Ethiopia [41]. Such variations may be attributed to different environmental and climatic factors, or even the type of fly trap used [11, 41, 42, 43]. In relation to disease epidemiology, the higher number of biting flies captured in Marsabit is related to the observations of higher *Surra* prevalence in Marsabit compared to Isiolo. However, a more comprehensive study is required to fully elucidate this in terms of linking fly infectivity with camel infections.

High fly catches in the present study were recorded in traps placed adjacent to water pans and ponds, confirming that this environment is very suitable for biting fly infestation. Most tabanids are known to lay their eggs around the edges of ponds in the moist mud. In the dry season sampling, the seasonal rivers had dried up, hence the secluded watering ponds provided a conducive environment for the flies to lay their eggs, as was observed in Bubisa, where high numbers of biting flies were recorded during the dry season sampling. It is naturally expected that camel keepers would avoid grazing their camels in areas with a high population of biting flies to avoid infection and nuisance caused by the flies. Indeed, this is one of the coping mechanisms employed by camel keepers in the study setting to prevent *Surra* [34].

Limited species diversity was observed in both the rainy and the dry season, perhaps due to the hot and dry environmental conditions in the study area. Comparable fly diversity has been previously recorded in Marsabit county [44]. In contrast, a wider diversity of fly species is generally reported in forested areas [45]. Camel ked flies (*H. camelina*), which were visually observed on the camels in abundance during blood sampling, were not captured in the traps*. Camel flies* are ectoparasites that attach firmly to the hairs on the camel's skin using tarsal claws to enable them to move together with the camels. Therefore, their reduced flight ability may contribute to their inability to fly into the sampling traps. The camel has been shown to provide up to 60% of *H. camelina* bloodmeals [44] and is thus an important potential vector for *T. evansi*. In the present study, two species identified, viz., *Tabanus taeniola* and *A. agrestis*, comprised the largest proportion of the biting flies captured. Working in Marsabit over a 3-year period, Oyieke [46] captured three species of *Tabanus*, namely *T. taeniola*, *Tabanus leucostomus*, and *Tabanus atrimanus*, of which only the first was captured in the present study. *A. agrestis*, which was the most abundant species captured in the present study, has previously been confirmed as a competent vector of both *T. congolense* and *T. evansi* [41, 47]. This would indicate that in addition to *T. evansi*, this species would also have a role to play in *T. congolense* transmission. Infection of camels with *T. congolense* in non-tsetse infected areas has often been attributed to the transit of animals through a tsetse-infested area but this may not always be the case. Additional work to link biting flies to the mechanical transmission of *T. congolense* in this area may help to fully elucidate the situation.

The seasonal variations of specific abundances that were observed in this study follow a predictable pattern, showing an increase during the wet season. There are reported correlations between increased biting fly numbers in the rainy season and the seasonal outbreaks of *T. evansi* infections in various tropical areas [21, 48]. In general, all species that were captured in the dry season were also captured in the wet season, albeit in higher numbers. Species such as *H. camelina* and *S. calcitrans* have been reported to be found all year round, and indeed in a complementary work that was carried out within this project, about 16% (*n* = 340) of respondents indicated that *Surra* vectors are found all year round [49]. To fully define the effect of vector abundance, the incorporation of additional epidemiological factors is required to relate vector presence/abundance to the risk of *T. evansi* transmission.

## 5. Conclusion

The present study has demonstrated geographic, seasonal, and between-herd variations in *Surra* and vector prevalence. Spatial–temporal variations in trypanosome prevalence observed in this study were consistent with those of vector distribution recorded during the rainy and dry seasons. The study identified for the first time co-infections of camels in North Eastern Kenya by *Trypanozoon (T. evansi* or *T. b. brucei*) and *T. vivax*, which necessitates targeted therapeutic management of *Surra* that hitherto largely relies on the use of quinapyramine sulphate and chloride. The findings have further revealed that cattle and goats may not be significant reservoirs of *T. evansi* in the region, however, this need to be validated using more sensitive and specific molecular screening methods. The study used parasitological screening methods (microscopy) that have relatively low sensitivity and specificity, the actual *Surra* prevalence is likely to be higher in the study area. This study identified some biting flies, which may be probable vectors of *Surra* in Marsabit and Isiolo counties, as a contribution toward understanding disease epidemiology. This information may form an important component in developing an integrated disease control strategy for this region.

## Figures and Tables

**Table 1:** Prevalence of trypanosome infection in the different sites at the beginning of the dry season, peak dry season, and during the rainy season.

Study site	Rainy season		Peak of the dry season		Beginning of the dry season	
	Number of camels sampled	Prevalence (%)	Number of camels sampled	Prevalence (%)	Number of camels sampled	Prevalence (%)
Isiolo county						
Ngaremara	147	4.1	160	1.3	57	0.0
Kinna	72	0.0	123	0.0	104	1.9
Kula Mawe	104	8.7	100	1.0	96	12.5
LMD	101	0.0	138	0.0	146	0.0
Marsabit county						
Bubisa	100	4.0	123	2.4	100	2.0
Turbi	100	0.0	123	4.1	100	4.0
Loglogo	100	11.0	112	11.6	60	36.7
Laisamis	100	4.0	200	6.5	181	9.9
Total	824	4.1	1,079	3.4	844	7.1

**Table 2:** Herd-level prevalence of trypanosome infections in the blood of camels sampled across different sites in Isiolo and Marsabit counties.

Study site	Rainy season		Peak of the dry season		Start of the dry season	
	Number of sampled herds	Highest prevalence recorded (%)	Number of sampled herds	Highest prevalence recorded (%)	Number of sampled herds	Highest prevalence recorded (%)
Isiolo county						
Ngaremara	9	14.3	7	5.0	3	19.3
Kinna	2	0.0	4	0.0	5	4.5
Kula Mawe	9	60.0	5	5.0	4	0.0
LMD	3	0.0	2	0.0	3	0.0
Marsabit county						
Bubisa	8	33.3	15	17.0	9	12.5
Turbi	7	0.0	11	38.0	10	15.0
Loglogo	6	31.3	10	16.0	10	71.4
Laisamis	8	22.2	5	33.0	5	21.4
Total	52		59		49	

**Table 3:** Mean packed cell volume of infected and non-infected camels by age category.

Trypanosome infection	Age category	Wet season (mean ± SD)	Peak of dry season (mean ± SD)	Beginning of dry season (mean ± SD)
Infected	Calf	17.4 ± 2.4	20.6 ± 3.0	19.2 ± 2.8
	Young adult	22.3 ± 2.1	17.3 ± 2.4	19.6 ± 3.7
	Adult	20.0 ± 3.4	20.8 ± 3.1	20.8 ± 5.2
	Total	19.6 ± 3.5	20.1 ± 3.2	20.1 ± 4.3
Non-infected	Calf	24.4 ± 3.2	26.1 ± 3.6	26.2 ± 4.0
	Young adult	25.2 ± 3.0	25.5 ± 3.7	25.5 ± 4.6
	Adult	25.4 ± 3.5	25.3 ± 3.9	26.0 ± 3.8
	Total	25.0 ± 3.5	25.5 ± 3.8	25.9 ± 4.0
*p*-values		<0.001	<0.001	<0.001

**Table 4:** Comparison of proportion of anemic and non-anemic camels in the eight [8] sampling sites in the wet season sampling.

Sampling site	Number sampled (*n*)	Anemic (PCV ≤ 23)	Proportion of camels anemic (%)	Non-anemic (PCV > 23)	Proportion of camels non-anemic (%)
Marsabit county					
Turbi	100	28	28.0	72	72.0
Bubisa	100	13	13.0	87	87.0
Loglogo	100	46	46.0	54	54.0
Laisamis	100	26	26.0	74	74.0
Total	400	113	28.3	287	71.8
Isiolo county					
Ngaremara	147	52	35.4	95	64.6
LMD	101	24	23.8	77	76.2
Kula Mawe	103	31	30.1	72	69.9
Kinna	72	15	20.8	57	79.2
Total	423	122	28.8	301	71.2
Overall	823	235	28.6	588	71.5

**Table 5:** Dry season counts of biting flies in Marsabit and Isiolo.

County	Area	Flies captured			
		*Tabanus*	*Philoliche*	*Chrysops*	*Stomoxys*
Marsabit	Bubisa	263	—	—	2
	Turbi	10	—	—	—
	Loglogo	49	4	—	—
	Laisamis	135	—	—	1
Isiolo	LMD	16	—	—	11
	Ngaremara	49	1	—	10
	Kinna	62	1	—	8
	Kula Mawe	6	7	2	10

**Table 6:** Wet season counts of biting flies in Marsabit and Isiolo.

County	Area	Flies captured			
		*Tabanus*	*Philoliche*	*Chrysops*	*Stomoxys*
Marsabit	Bubisa	9	—	—	39
	Turbi	17	—	1	6
	Loglogo	49	3	—	61
	Laisamis	37	1	—	29
Isiolo	LMD	5	14	—	29
	Ngaremara	97	8	8	34
	Kinna	38	8	—	11
	Kula Mawe	24	33	—	9

## Data Availability

Data files used in the paper are available from the Mendeley data and can be accessed here: https://data.mendeley.com/datasets/b64fg8zbyg/1.

## Supplementary Materials

Supplementary Materials Table A Disaggregation of screened camels by sex and age categories in the study sites. Table B Proportion of anemic and non-anemic cattle across the eight [8] sampling sites during the rainy season.
