# Supplementary material for: Spatial–Temporal Variations in Parasitological Prevalence and Host-Related Risk Factors of Camel Trypanosomiasis and Its Vectors in North Eastern Kenya: A Repeated Cross-Sectional Study
Source: J Parasitol Res. 2023 Apr 28;2023:7218073. doi: 10.1155/2023/7218073 (PMC10162873; doi:10.1155/2023/7218073)
Supplement: Supplementary Materials — Table A Disaggregation of screened camels by sex and age categories in the study sites. Table B Proportion of anemic and non-anemic cattle across the eight [8] sampling sites during the rainy season. [file 7218073.f1.docx]

**Supplementary files detailing different characteristics of camels, cattle and goats screened in the study**

**Table A.** **Disaggregation of screened camels by sex and age categories in the study sites**

| **Study site** | **Number of camels** | **Percent (%)** | **Females** | **Males** | **Adults (>5years)** | **Young adults**  **(2-5years)** | **Calves (<2years)** |
| --- | --- | --- | --- | --- | --- | --- | --- |
| **Isiolo county-Start of dry season** | | | | | | | |
| 1. Ngaremara | 96 | 11.6 | 72 | 25 | 53 | 14 | 31 |
| 1. Kinna | 105 | 12.4 | 78 | 27 | 33 | 34 | 37 |
| 1. Kula Mawe | 57 | 6.7 | 45 | 11 | 35 | 7 | 15 |
| 1. LMD | 146 | 17.2 | 110 | 33 | 57 | 15 | 71 |
| **Marsabit county-Start of dry season** | | | | | | | |
| 1. Bubisa | 100 | 11.8 | 73 | 27 | 71 | 19 | 10 |
| 1. Turbi | 100 | 11.8 | 64 | 36 | 64 | 20 | 16 |
| 1. Logologo | 60 | 7.1 | 44 | 16 | 34 | 15 | 11 |
| 1. Laisamis | 181 | 21.4 | 110 | 71 | 75 | 36 | 70 |
| **Total** | **847** | **100.0** | **596** | **246** | **422** | **160** | **261** |
| **Isiolo county-Peak of dry season** | | | | | | | |
| 1. Ngaremara | 160 | 14.8 | 122 | 38 | 84 | 24 | 51 |
| 1. Kinna | 123 | 11.4 | 101 | 22 | 72 | 14 | 37 |
| 1. Kula Mawe | 100 | 9.3 | 91 | 9 | 77 | 7 | 16 |
| 1. LMD | 138 | 12.8 | 104 | 34 | 60 | 22 | 56 |
| **Marsabit county-Peak of dry season** | | | | | | | |
| 1. Bubisa | 123 | 11.4 | 102 | 21 | 91 | 18 | 14 |
| 1. Turbi | 123 | 11.4 | 111 | 10 | 113 | 0 | 10 |
| 1. Logologo | 112 | 10.4 | 90 | 22 | 60 | 20 | 32 |
| 1. Laisamis | 200 | 18.5 | 139 | 61 | 101 | 34 | 65 |
| **Total** | **1079** | **100.0** | **860** | **217** | **658** | **281** | **139** |
| **Isiolo county-Wet season** | | | | | | | |
| 1. Ngaremara | 147 | 17.8 | 102 | 45 | 74 | 27 | 46 |
| 1. Kinna | 72 | 8.7 | 64 | 8 | 49 | 8 | 15 |
| 1. Kula Mawe | 104 | 12.6 | 85 | 19 | 62 | 4 | 38 |
| 1. LMD | 101 | 12.3 | 89 | 12 | 54 | 13 | 34 |
| **Marsabit county-Wet season** | | | | | | | |
| 1. Bubisa | 100 | 12.1 | 90 | 10 | 90 | 3 | 7 |
| 1. Turbi | 100 | 12.1 | 81 | 19 | 77 | 1 | 22 |
| 1. Logologo | 100 | 12.1 | 77 | 23 | 71 | 10 | 19 |
| 1. Laisamis | 100 | 12.1 | 71 | 29 | 59 | 8 | 33 |
| **Total** | **824** | **100** | **659** | **165** | **536** | **74** | **214** |

**Table B. Proportion of anemic and non-anemic cattle across the eight (8) sampling sites during the rainy season**

| **Sampling site** | **Number sampled (n)** | **Anaemic (PCV≤25)**  **(n)** | **Proportion of anaemic camels** | **Non-anaemic (PCV>25)**  **(n)** | **Proportion of non-anaemic camels** |
| --- | --- | --- | --- | --- | --- |
| **Marsabit county** | | | | | |
| Turbi | 50 | 4 | 1.0% | 46 | 11.4% |
| Bubisa | 50 | 4 | 1.0% | 46 | 11.4% |
| Loglogo | 50 | 9 | 2.2% | 41 | 10.1% |
| Laisamis | 50 | 12 | 3.0% | 38 | 9.4% |
| **Total** | **200** | **29** | **7.2%** | **171** | **42.2%** |
| **Isiolo county** | | | | | |
| Ngaremara | 54 | 9 | 2.2% | 45 | 11.1% |
| LMD | 51 | 14 | 3.5% | 37 | 9.1% |
| Kula Mawe | 50 | 12 | 3.0% | 38 | 9.4% |
| Kinna | 50 | 11 | 2.7% | 39 | 9.6% |
| **Total** | **205** | **46** | **11.4%** | **159** | **39.3%** |
| Overall | 405 | 75 | 18.5% | 330 | 81.5% |
